# Differential Translocation of Host Cellular Materials into the *Chlamydia trachomatis* Inclusion Lumen during Chemical Fixation

**DOI:** 10.1371/journal.pone.0139153

**Published:** 2015-10-01

**Authors:** Marcela Kokes, Raphael H. Valdivia

**Affiliations:** Department of Molecular Genetics and Microbiology and Center for the Genomics of Microbial Systems, Duke University Medical Center, Durham, North Carolina, United States of America; University of California, San Francisco, University of California, Berkeley, and the Children's Hospital Oakland Research Institute, UNITED STATES

## Abstract

*Chlamydia trachomatis* manipulates host cellular pathways to ensure its proliferation and survival. Translocation of host materials into the pathogenic vacuole (termed ‘inclusion’) may facilitate nutrient acquisition and various organelles have been observed within the inclusion, including lipid droplets, peroxisomes, multivesicular body components, and membranes of the endoplasmic reticulum (ER). However, few of these processes have been documented in living cells. Here, we survey the localization of a broad panel of subcellular elements and find ER, mitochondria, and inclusion membranes within the inclusion lumen of fixed cells. However, we see little evidence of intraluminal localization of these organelles in live inclusions. Using time-lapse video microscopy we document ER marker translocation into the inclusion lumen during chemical fixation. These intra-inclusion ER elements resist a variety of post-fixation manipulations and are detectable via immunofluorescence microscopy. We speculate that the localization of a subset of organelles may be exaggerated during fixation. Finally, we find similar structures within the pathogenic vacuole of *Coxiella burnetti* infected cells, suggesting that fixation-induced translocation of cellular materials may occur into the vacuole of a range of intracellular pathogens.

## Introduction

*Chlamydia trachomatis* is a human pathogen of global significance–it is the most common sexually-transmitted bacterial pathogen and the leading cause of preventable blindness worldwide [1]. During infection of mammalian cells, bacteria remain sequestered within a membrane-bound compartment termed an inclusion. The inclusion is fundamental to the intracellular lifestyle of *C*. *trachomatis* as it represents a first line of defense against immune surveillance and anti-microbial effectors [2]. In addition, as an obligate intracellular pathogen, *C*. *trachomatis* requires vital nutrients obtained from its infected host cell (reviewed in [3]) and interactions between the inclusion and host cellular components play an important role in this process (reviewed in [4]).

Many subcellular organelles reside in close proximity to the inclusion, some of which have been reported to interact intimately with inclusions (reviewed in [4]). In a well-characterized example, endoplasmic reticulum (ER) tubules closely appose to *C*. *trachomatis* inclusion membranes [5,6] to form membrane contact sites that facilitate the transfer of lipids directly between the ER and the pathogenic vacuole [7,8]. The Golgi apparatus fragments into mini-stacks [9] in an actin and bacterial effector-dependent manner [10] which closely surround the inclusion [11,12]. Lipid droplets are recruited to the inclusion periphery [13] and can translocate into the inclusion lumen [14] while lysosomes closely surround the inclusion to presumably enhance the ability of bacteria to acquire amino acids from degraded proteins [15]. Multivesicular bodies (MVBs) are enriched at the inclusion periphery and inhibition of MVB biogenesis can disrupt *C*. *trachomatis* lipid uptake [16,17]. Recycling endosomes closely associate with the inclusion [18–20] but are thought to interact with the inclusion in a fusion-inhibited state [19,21]. Mitochondria localize around the inclusion of some *Chlamydia* species [6,22], and mitochondrial protein import is important for replication of *C*. *caviae* [23].

In several instances, interactions with the inclusion extend beyond recruitment to the periphery and entire organelles translocate into the inclusion lumen. Briefly, protein markers of sphingolipids [11], cholesterol [24], lipid droplets[14], and the ER [25] have been reported within the inclusion of living cells. Components of the ER [25,26], MVBs [16], and peroxisomes [27], have been observed within the inclusion of fixed cells, as well as Rab14 [28], apoA-1 and phosphatidylcholine [29] and proteins that interact with acyl-CoA or each other including ASCL3, ACBD6, and ZNF23 [30]. Additionally, some subcellular components and markers such as MVBs [31] or phosphatidylcholine [12] have been only observed in inclusions within only fixed cells.

In this study, we sought to characterize organelle interactions with the inclusion by systematically surveying organelle markers for localization within infected cells. We found a subset of the many inclusion-proximal subcellular elements within inclusions, including components of the ER, mitochondria, and markers of the inclusion membrane. We document the striking translocation of ER markers into the inclusion lumen during the process of chemical fixation. These internalized structures persisted through the process of sample processing for indirect immunofluorescence, indicating that the localization of a subset of organelles to the inclusion may be exaggerated by the fixation process rather than accurately reflecting organelle localization prior to manipulation. Finally, we observed similar structures within the pathogenic vacuole of *Coxiella burnettii*-infected cells, suggesting that fixation-induced translocation of subcellular elements may constitute a more widespread phenomenon in the study of intracellular pathogens residing within large vacuoles.

## Results

### A Subset of Inclusion-Proximal Subcellular Organelles Localize to the Lumen of Inclusions

**Fig 1:**
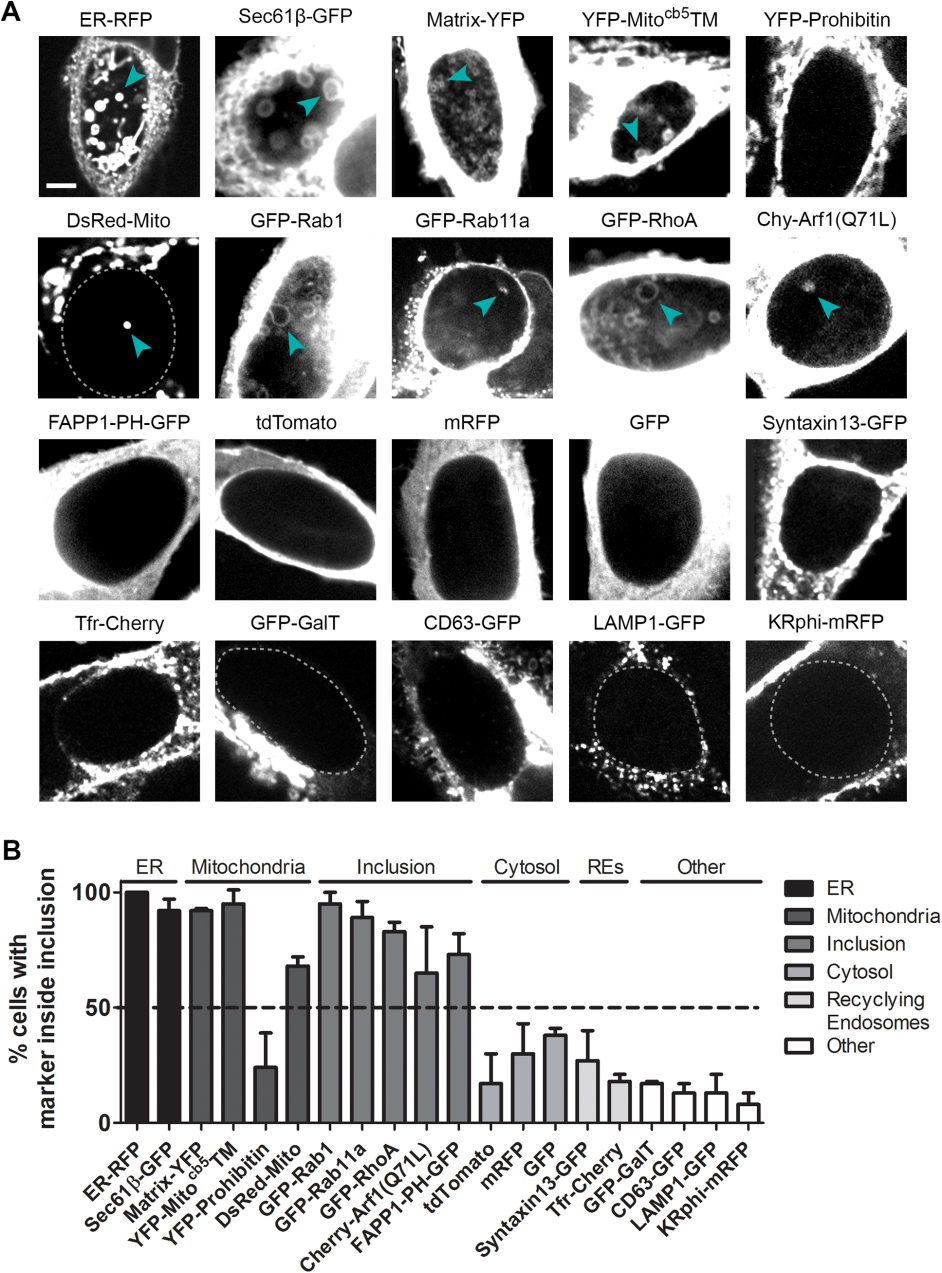
Mitochondria, ER, and inclusion membranes are found within the lumen of *C*. *trachomatis* inclusions. HeLa cells were infected with *C*. *trachomatis* LGV L2, transfected with the indicated plasmids, and fixed at 30 hpi for serial spinning disk laser confocal analysis. Note the presence of markers of the ER, mitochondrial matrix and outer membranes, and inclusion membranes within the inclusion lumen (A, cyan arrowheads). Images portray a single z-section from the center of an inclusion, and inclusions are visually identified as large black centered ovals or outlined with a dashed line. Cellular-localized markers appear saturated because material within the inclusion was often significantly dimmer. (B) The frequency of internalized structures within the entire 3D space of each inclusion was assessed. Plasmids are categorized as markers of the ER, mitochondria, inclusion, cytosol, recycling endosomes, or other as indicated. Within the other category, GFP-GalT localizes to the Golgi, CD63-GFP to MVBs, LAMP1-GFP to lysosomes, and KRphi-mRFP to the plasma membrane. A dashed line at 50% distinguishes between high and low frequencies of intraluminal structures within inclusions. 12–20 inclusions were assessed in each experiment, and the mean ± SEM for three independent experiments is shown. Scale bar represents 5 μm.

We surveyed interactions between host organelles and mid-to-late cycle inclusions by expressing a panel of fluorescent protein-tagged markers of various subcellular organelles in cells infected for 30 hr (Fig 1A). We fixed cells and quantified the frequency of cells with fluorescent material in the inclusion lumen (Fig 1B). To enhance our ability to accurately define the inclusion edge in three dimensions without interference from light above and below each plane of focus, we used confocal rather than widefield microscopy. Furthermore, since most markers had much lower intensity within the inclusion compared to cellular structures, we used spinning disk rather than laser scanning confocal microscopy to reduce photobleaching while imaging cells in three dimensions.

Of all intracellular compartments tested, we found markers of the ER lumen and membrane, mitochondrial matrix and outer membrane [32], and proteins which, in addition to differentially localizing to a range of subcellular compartments, also associate with the inclusion (FAPP1-PH-GFP is a marker of phosphatidylinositol 4-phosphates [33] which are enriched on the inclusion [34]) [35,36] within a significant proportion (over 50%) of inclusions (Fig 1B) appearing as bleb-like structures (Fig 1A, arrowheads). Notably, we found markers of the cytosol and other inclusion-proximal subcellular organelles including recycling endosomes, MVBs, the Golgi apparatus, and lysosomes [9,12,16,18,21,31] also as bleb-like structures within only approximately one-quarter of inclusions, but did not observe a marker of the more distal plasma membrane [37] within inclusions. These findings reveal a selectivity to which inclusion-proximal subcellular elements are found within inclusions in fixed cells.

### ER Components Appear as Large Structures inside the Inclusion of Fixed but Not Living Cells

**Fig 2:**
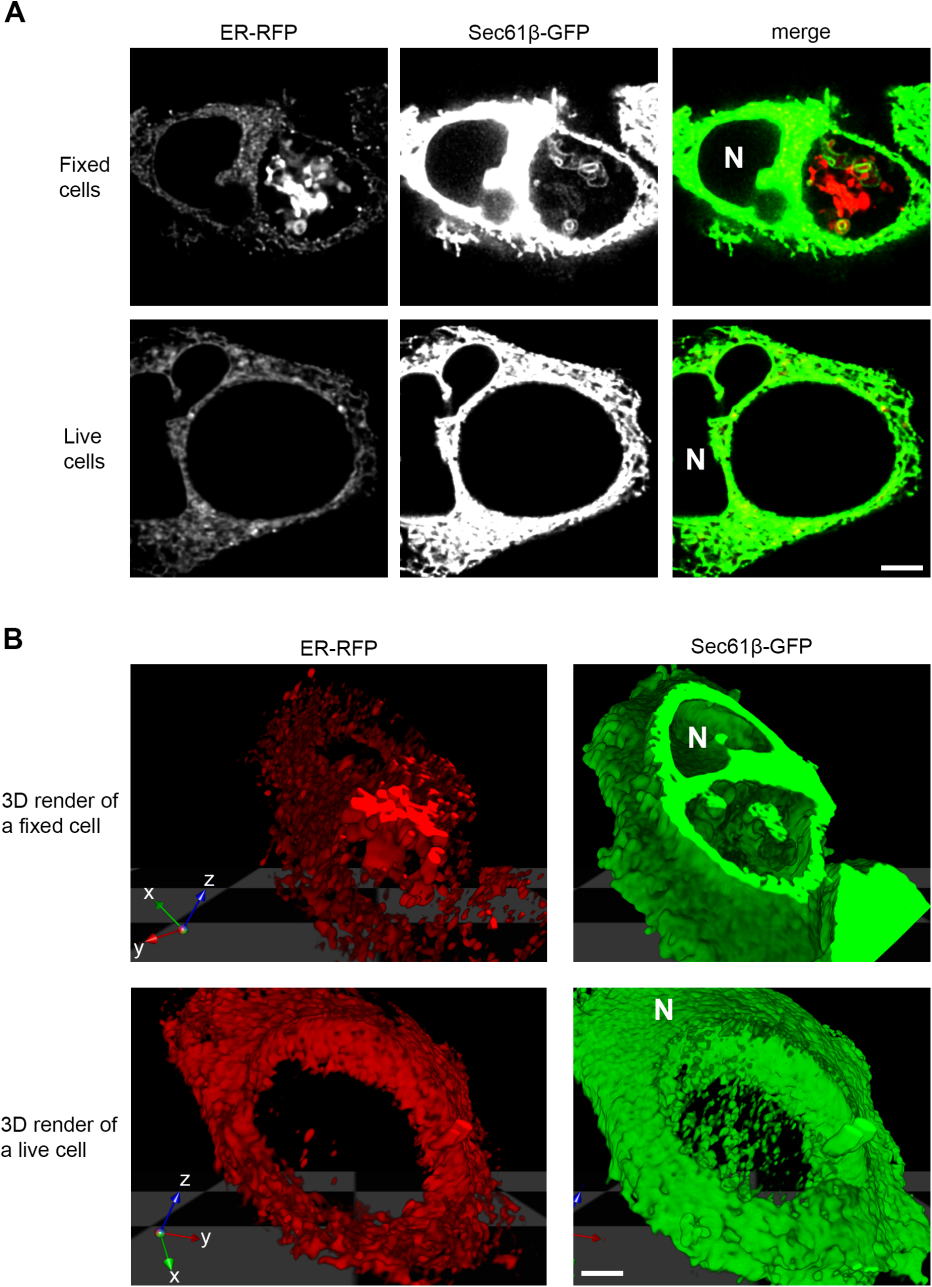
ER markers reveal expansive structures within the inclusion lumen of fixed but not living cells. HeLa cells were infected with *C*. *trachomatis* LGV L2, co-transfected with ER-RFP (red) and Sec61β-GFP (green) and at 30 hpi were either fixed or imaged in three dimensions while living. Images were acquired with a laser scanning confocal microscope. (A) A single xy micrograph towards the center of a cell. See S1 and S2 Movies for a progression of xy micrographs along the z-axis. (B) Images were used to render volumes in 3D. 3D render of a fixed cell is limited in the z-axis to allow viewing within the inclusion. See S3–S6 Movies for QuickTime Virtual Reality files to rotate and view these 3D volumes. Note the presence of an expansive network of material within the inclusion lumen of fixed cells. N marks the nucleus. Scale bars represent 5 μm.

The protein markers that were most notably visible within the lumen of inclusions, such as ER-RFP and Sec61β-GFP, typically formed an expansive network of large blebs and tubules within much of the three-dimensional space of the inclusion lumen in fixed cells (Fig 2A and 2B, top panel, S1, S3, and S4 Movies). However, within the limits of our experimental design and number of cells analyzed, we were unable to detect these structures within living infected cells (Fig 2A and 2B, bottom panel, S2, S5 and S6 Movies).

### Chemical Fixation Induces ER Internalization into the Inclusion Lumen

**Fig 3:**
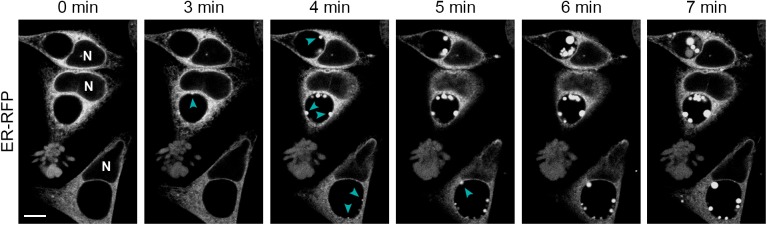
ER-RFP translocates into the inclusion lumen during chemical fixation. HeLa cells were infected with *C*. *trachomatis* LGV L2 and transfected with ER-RFP. At 30 hpi, living cells were placed in 4% paraformaldehyde and imaged over time with a laser scanning confocal microscope. Note the genesis of ER-RFP blebs into the inclusions lumen (cyan arrowheads) during fixation. See S7 Movie for a time-lapse video including more cells in a larger field of view. N marks the nuclei. Scale bar represents 10 μm.

Because of the differences in ER localization patterns between living and fixed cells, we considered the possibility that the process of fixation exaggerates the degree of translocation of this organelle into the inclusion lumen. To test this, we used laser scanning confocal microscopy on living infected cells to monitor changes to ER-RFP localization during paraformaldehyde fixation (Fig 3 and S7 Movie). Within minutes of the addition of fixative, we observed the formation of blebs of ER-RFP material expanding into the inclusion lumen. These blebs appeared at random sites along the inclusion periphery and enlarged over time as new sites of inward blebbing (Fig 3, arrowheads) emerged. Many blebs remained attached to the inclusion edge but some appeared to detach into the center of the inclusion. By ten minutes, most new ER blebbing and expansion had stopped and existing structures varied in fluorescence intensity. We also noted the formation of a few smaller and less distinct bleb-like aggregates of ER-RFP in other areas of the cell, mostly at cell edges. These findings demonstrate that chemical fixation can induce a dramatic translocation of ER material into the inclusion lumen.

### Chemical Fixation Influences the Degree to Which Organelles Are Observed within the Inclusion

**Fig 4:**
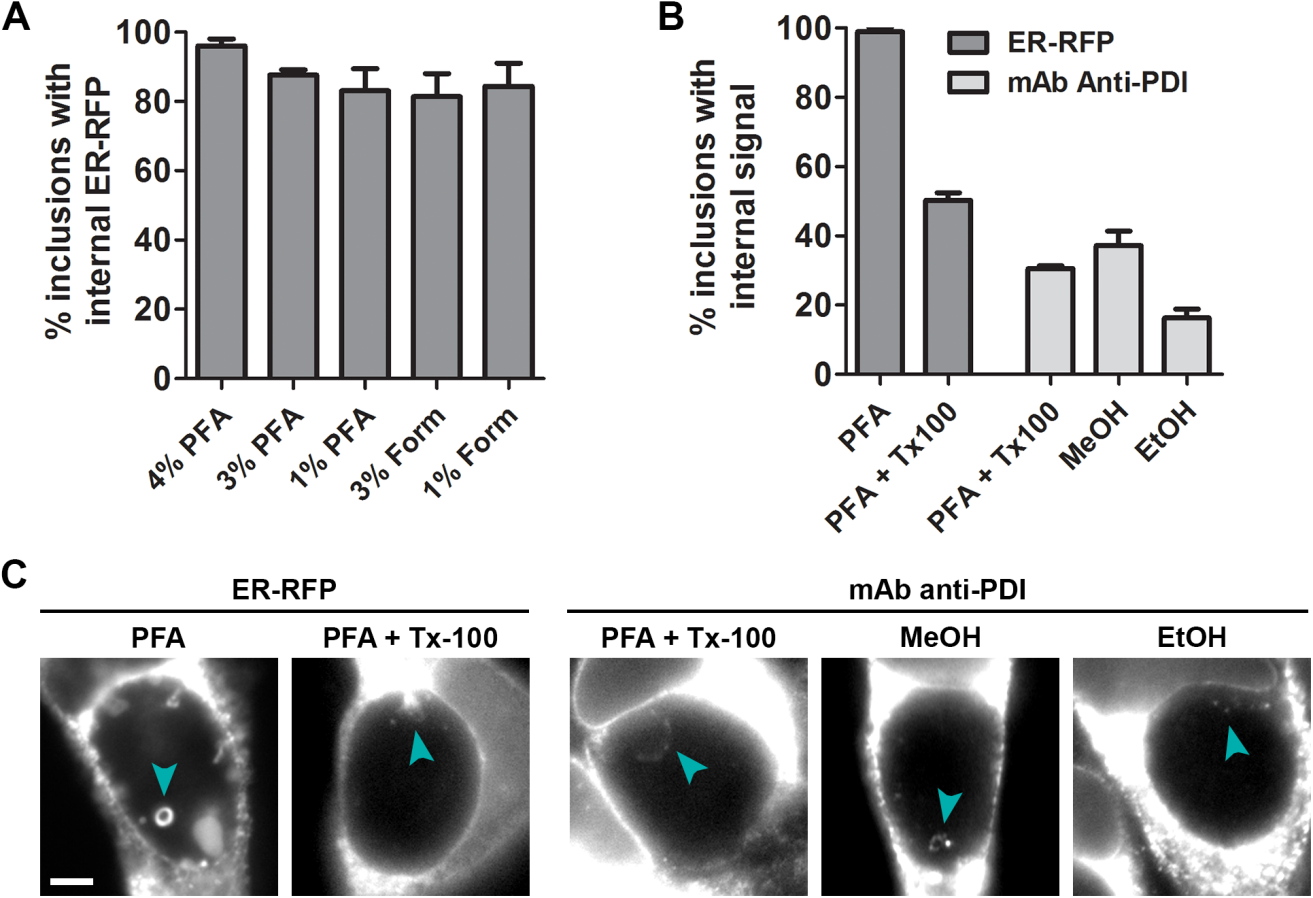
ER structures are detected via immunofluorescence microscopy within inclusions after both formaldehyde and alcohol-based chemical fixation. HeLa cells were infected with *C*. *trachomatis* LGV L2. (A) Infected cells were transfected with ER-RFP, fixed at 30 hpi with different concentrations of either paraformaldehyde (PFA) or formaldehyde (Form), and assessed for the frequency of ER-RFP within inclusions. (B and C) Infected cells were transfected with ER-RFP as indicated. At 30 hpi, cells were fixed with PFA, methanol, or ethanol, and subsequently permeabilized with Triton X-100 (Tx100) as indicated. Treated cells were processed for immunofluorescence with an antibody to the ER protein PDI and assessed for the frequency of PDI-positive structures within inclusions. Scale bar represents 5 μm.

To assess whether the organelle internalization process can occur during various fixation techniques used previously in the study of host cellular material within *Chlamydia* inclusions [12,14,16,28,29], we quantified the frequency of ER-RFP structures in inclusions after fixation of infected cells (Fig 4A). Fixation with different concentrations of formaldehyde prepared from either paraformaldehyde or liquid formalin (which contains small amounts of methanol) resulted in similarly high frequencies of inclusions with internal ER-RFP structures, indicating that formaldehyde concentrations and trace levels of methanol in formalin do not affect the frequency of internalization. Since gluteraldehyde fixation as used for electron microscopy causes high levels of autofluorescence across the visible light spectrum [38], we could not reliably distinguish any fluorescent marker or dye tested from background fluorescence under these conditions (data not shown). Furthermore, we could not assess the frequency of ER-RFP structures within inclusions after methanol fixation since mRFP fluorescence was quenched by this treatment.

To compare the effect of denaturing fixatives such as alcohols to cross-linking fixatives such as aldehydes, we monitored ER internalization into the inclusion via immunofluorescence microscopy under different conditions. Because alcohol fixation simultaneously fixes and permeabilizes membranes, we first assessed how well ER-RFP intra-inclusion structures resist detergent permeabilization. Using a range of conditions previously employed in the study of host cellular material within inclusions [12,14,16], we found that ER-RFP structures persisted at a reduced number per inclusion (Fig 4C) and typically in only half of all inclusions (Fig 4B and S1 Fig). We next performed immunofluorescence on paraformaldehyde-fixed and detergent-permeabilized cells and compared with methanol or ethanol fixed/permeabilized cells using an antibody to the ER-resident protein PDI (Fig 4B and 4C). Notably, we observed PDI-positive structures under all conditions within one-quarter to one-half of inclusions. These findings suggest that both formaldehyde and alcohol-based chemical fixation result in the translocation of endogenous ER materials into the inclusion lumen.

### ER Material Is Internalized into the Vacuole of Another Intracellular Pathogen

**Fig 5:**
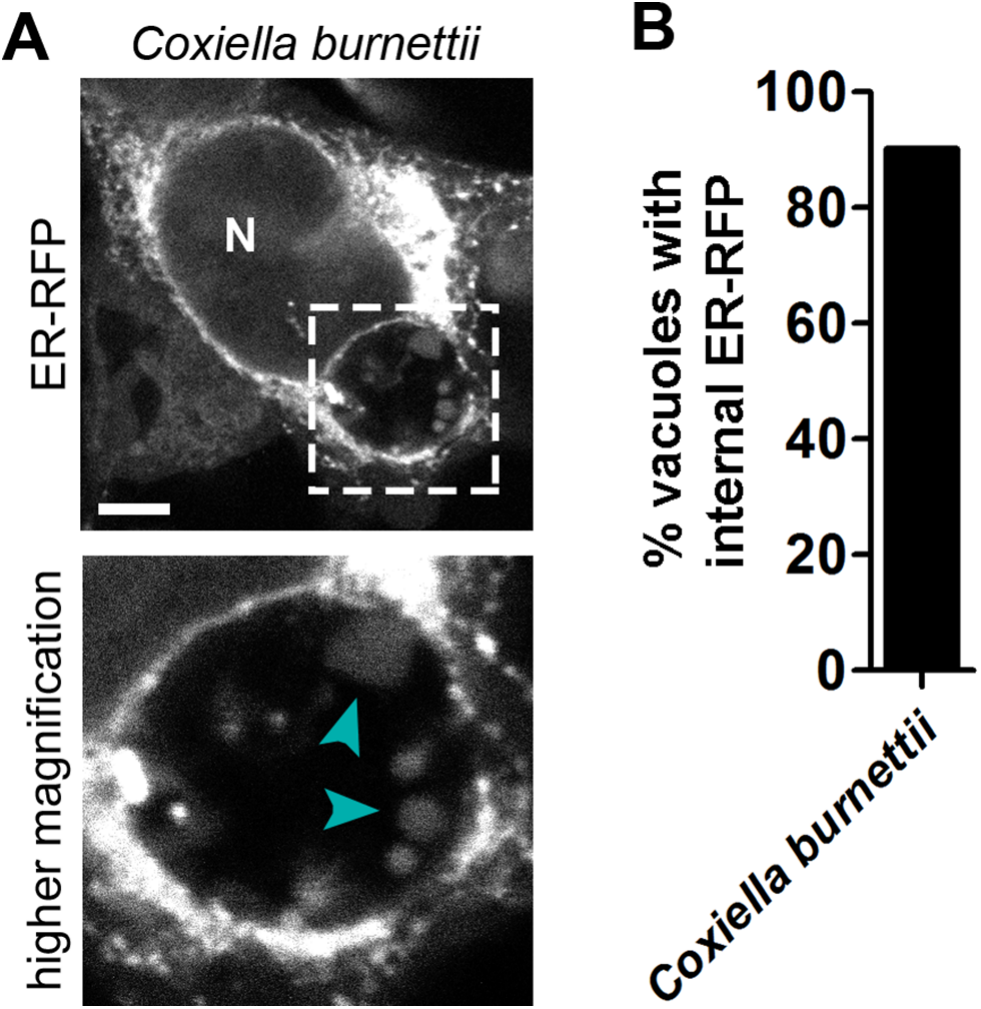
ER-RFP within the pathogenic vacuole of *Coxiella burnettii*. HeLa cells were infected with *Coxiella burnettii*, transfected with ER-RFP, and fixed at 52 hpi. Note the presence of ER-RFP structures with in the lumen of the pathogenic vacuole (cyan arrowheads) similar to those within *Chlamydia trachomatis* inclusions. N indicates the nucleus and a dashed line outlines a region of higher magnification. Scale bar represents 5 μm.

The *Chlamydia* inclusion is an unusually spacious organelle and the luminal space is largely devoid of electron-dense material compared to the host cytoplasm as assessed by transmission electron microscopy [39]. We speculated that this spacious nature may be conducive to fixation-induced translocation of materials that might otherwise not occur elsewhere in the cell. To assess this, we asked whether another similarly spacious pathogenic vacuole occupied by *Coxiella burnettii* (reviewed in [40]) would display similar internalized structures. When we transfected *Coxiella burnettii*-infected cells with ER-RFP and fixed after 52 hr of infection, we found ER-RFP structures within the lumen of the pathogenic vacuole with similar appearance and frequency (90%) as those found within *Chlamydia* inclusions (Fig 5).

## Discussion

In this study, we report that fluorescently tagged markers of the ER, mitochondria, and inclusion membranes are readily observed within the lumen of fixed *Chlamydia trachomatis* inclusions. However this may not be an accurate reflection of *in vivo* processes as we determined that chemical fixation can induce the translocation of ER components into the inclusion. These internalized structures resist many common immunostaining procedures and were detected by immunofluorescence microscopy, indicating that select cellular materials detected within the inclusions of fixed cells may overestimate the true localization in living cells.

Our findings indicate that fixation-induced internalization into the inclusion lumen is selective amongst subcellular components that reside in close proximity to the inclusion. We routinely found markers of the inclusion membrane, ER lumen, ER membrane, mitochondrial matrix and mitochondrial outer membrane within the inclusion lumen of fixed cells–organelles which all closely associate with the inclusion. However, markers of other inclusion-proximal subcellular elements, including the Golgi, recycling endosomes, lysosomes, and the cytosol, did not. Similarly, we did not observe a marker of the more distal plasma membrane within inclusions. As lipids are directly transferred between the ER and inclusion at membrane-contact sites between the two [7,8], we speculate that fixation-induced internalization may occur specifically at inclusion-organelle interaction areas of direct material transfer. Indeed, we infrequently observed what appeared to be the translocation of ER markers across the plasma membrane outward from cells during fixation, and membrane-contact sites between the ER and the plasma membrane have been reported (reviewed in [41]). Some organelles have been suggested to directly transfer into the inclusion, including lipid droplets [14] and peroxisomes [27], with the translocation of lipid droplets (14) and ER (25) confirmed in living cells. In general, it remains unclear how fixation-induced translocations reflect *bona fide* interactions with the inclusion, although it is possible that fixation enhances the frequency of *bona fide* translocation or interaction events.

Methanol and ethanol disrupt hydrophobic and hydrogen bonding which denatures proteins, and are sometimes used to fix samples for immunofluorescence microscopy due to ease of use, but can shrink and distort tissues and cells [42]. Formaldehyde reacts with and crosslinks various reactive groups of biological molecules, including proteins, DNA, and sugars. It is routinely used to preserve cellular architecture and the spatial relationships of proteins in cell and tissues (reviewed in [42,43]). However, there are instances where chemical or aldehyde fixation alters rather than preserves cellular structures. A prominent example is the mesosome, which was thought to be a separate intracellular organelle in bacteria until it was shown to be an artifact of chemical fixation [44] of exaggerated invaginations from the cell membrane [45]. There is also precedence for fixation-induced membrane blebbing in mammalian cells. Exposing cell monolayers to low concentrations of paraformaldehyde causes the release of vesicles from the plasma membrane [46]. These vesicles range in size from 0.5 μm to 15 μm and appear at cell edges similarly to the infrequent blebbing of ER-RFP that we observed during fixation. The kinetics of large membrane blebs forming outwardly from cells fixed under standard paraformaldehyde concentrations [42] are similar to that of structures we observed forming within inclusion lumens and may share the same mechanism of genesis.

By transmission electron microscopy, the inclusion lumen appears spacious with large distances between bacteria particularly in the center, and except for glycogen, is largely devoid of electron-dense material [22,39], particularly in comparison to the host cell cytosol. This relatively empty lumenal space could be particularly conducive to the formation of formaldehyde-induced blebs, just as is the extracellular milieu. Consistent with this model, we also observed ER-RFP structures within fixed pathogenic vacuoles of *Coxiella burnettii* at 52 hpi–a time when this vacuole appears similarly spacious by electron microscopy (reviewed in [40]).

Our findings indicate that the process of chemical fixation can lead to the accumulation of subcellular components in the lumen of the pathogenic vacuole from two intracellular pathogens–*Chlamydia trachomatis* and *Coxiella burnettii*. The discrepancy between the relative efficiency with which ER membranes are found in the lumen of inclusions between fixed and live cells suggest that caution should be exercised when interpreting events observed in fixed cells. As imaging technologies surpass the diffraction-limit of light and capture images at super-resolution, sub-micron level differences in protein localization and aggregation are becoming apparent between chemically fixed and living cells [47]. Alternative fixation techniques that better preserve structures, such cryofixation should also reduce subcellular distortions [48] as has been used to confirm the presence of ER fragments within inclusions [25]. Fixation is unlikely to perfectly preserve the internal architecture of cells and thus observing subcellular components in living, intact cells should remain the gold standard when assessing the significance of any observed interactions. Ideally, by assessing the interaction between the inclusion and host organelles by a combination of multiple techniques, a more accurate picture of what occurs in unperturbed *C*. *trachomatis*-infected cells will emerge.

## Materials and Methods

### Cell Culture, *Chlamydia* Infections, Transfection, Antibodies and Plasmids

HeLa cells (ATCC CCL-2) were grown in high glucose DMEM supplemented with L-glutamine, sodium pyruvate (Gibco, Life Technologies) and 10% FBS (Mediatech, CellGro), at 37°C in a 5% CO_2_ humidified incubator. *C*. *trachomatis* LGV biovar L2 434/Bu [13] was propagated in Vero cells (ATCC CCL-81) and purified as previously described [49]. EB titers were determined by infecting Vero cell monolayers seeded in a 96 well plate. At 24 hpi cells were fixed and stained with anti-MOMP antibodies. Inclusion forming units (IFUs) were counted using a Cellomics ArrayScan automated fluorescence imaging system (Thermo Scientific). Cells were infected at an MOI of 1, synchronized by centrifugation (2,500 x g for 30 min at 10°C) onto HeLa cell monolayers, and incubated for 30 hr. As indicated, cells were transfected at the time of infection with jetPRIME (Polyplus transfection) according to manufacturer directions with a fresh media exchange after 4 hr. Antibody and plasmid sources: rabbit anti-*Chlamydia* MOMP (Kenneth Fields, University of Kentucky), mouse anti-PDI (Abcam ab2792) ER-RFP and DsRed-Mito (Richard Youle, NIH), Sec61β-GFP (Addgene 15108, Tom Rapoport, Harvard Medical School), GFP-GalT (Addgene 11929), Matrix-YFP, YFP-Mito^cb5^TM and YFP-Prohibitin (Jennifer Lippincott-Schwartz, Eunice Kennedy Shriver NICHD), GFP-Rab1 (Craig Roy, Yale), GFP-RhoA, CD63-GFP, and LAMP1-GFP (Soman Abraham, Duke University), Chy-Arf1(Q71L) and mRFP (Micheal Ehlers, Pfizer Neuroscience, formerly Duke University), FAPP1-PH-GFP (Tamas Balla, Eunice Kennedy Shriver NICHD), tdTomato (Marc Caron, Duke University), KRphi-mRFP (Addgene 17276, Sergio Grinstein, University of Toronto), GFP (pcDNA3.1-CT, Invitrogen, Life Technologies), Syntaxin13-GFP (William Trimble, University of Toronto).

### Imaging of Subcellular Organelles and Quantitation of Intraluminal Structures in Fixed Inclusions

HeLa cells grown on glass coverslips to 50% confluence were infected with *C*. *trachomatis* LGV L2 and transfected with the indicated (Fig 1) plasmids. At 30 hpi, cells were fixed with 4% paraformaldehyde (PFA) in PBS at pH 7.4 for 20 minutes at RT, incubated with 1 μg/mL Hoescht 33258 (Life Technologies) in PBS for 20 minutes at RT, mounted to slides in 5 μl SlowFade Gold (Life Technologies), and sealed with nail polish. Images were acquired using a Marianas system (Intelligent Imaging Innovations) equipped with an inverted microscope (Zeiss, Axio-Observer using a 100x 1.4 NA oil objective) and a Yokogawa spinning disk confocal unit (model CSU-22). All the hardware was controlled by SlideBook version 4.2 (Intelligent Imaging Innovations). Z-sections were acquired from above to below each cell, with optimal spacing between z-sections to meet the Nyquist resolution criterion. To assess the frequency of fluorescent blebs within inclusions, both the DNA staining signal (to define the inclusion and nucleus) and fluorescent protein signal in each individual z-section (approximately 30 per cell) from a z-stack of images of each inclusion were viewed with Slidebook version 4.2 or 5.5 (Intelligent Imaging Innovations). At least 12 inclusions for each marker in each independent experiment were assessed. Values from three independent experiments were averaged and standard errors were calculated. Calculations and graphs were prepared with Prism (GraphPad Software) and images were processed for display with Photoshop CS6 (Adobe).

### Comparison of Intraluminal Structures in Living and Fixed Cells

For fixed cells, HeLa cells were prepared as above. For living cells, HeLa cells grown on 35 mm #1.5 glass-bottom dishes (MatTek) to 50% confluence were infected with *C*. *trachomatis* LGV L2 and cotransfected with ER-RFP and Sec61β-GFP. At 30 hpi, cells were imaged in a phenol red-free DMEM HG (Gibco, Life Technologies) media supplemented with 10% FBS and 10 μM HEPES (Gibco, Life Technologies) in a humidified chamber maintaining 37°C and 5% CO_2_. Images were captured on a Leica SP5 laser scanning confocal inverted microscope equipped with a 100x 1.4 NA oil objective. Z-sections were acquired at optimal spacing to meet the Nyquist resolution criterion. Images were deconvolved using Huygens Essential (SVI) and processed with ImageJ (NIH) to create video.AVI files and Photoshop CS6 (Adobe) for presentation. To create 3D-rendered volumes, deconvolved images were further processed with Volocity (PerkinElmer), with the z-axis expanded three-fold to reduce flatness. 3D volumes of each channel acquired of living and fixed cells were exported into the QuickTime (Apple Inc.) Virtual Reality format for viewing (S3–S6 Movies).

### Time-Lapse Imaging of ER-RFP Translocation during Chemical Fixation

HeLa cells grown on 35 mm # 1.5 glass-bottom dishes (MatTek) to 50% confluence were infected with *C*. *trachomatis* LGV L2 and cotransfected with ER-RFP and Sec61β-GFP. At 30 hpi, cells were bathed in a phenol red-free DMEM HG (Gibco, Life Technologies) media supplemented with 10% FBS and 10 μM HEPES in a humidified chamber maintaining 37°C and 5% CO_2_, and 8% paraformaldehyde in PBS was added to a final concentration of 4%. Images were captured on a Leica SP5 laser scanning confocal inverted microscope equipped with a 63x 1.2 NA water objective. Images were acquired every 3.4 sec and the z-position was adjusted manually as needed to offset focal drift due to thermal changes. Images were compiled for display with ImageJ (NIH) and Photoshop CS6 (Adobe).

### Quantitation of ER-RFP Intraluminal Structures in Inclusions after Various Fixation and Permeabilization Conditions

HeLa cells grown on glass coverslips to 50% confluence were infected with *C*. *trachomatis* LGV L2 and transfected with ER-RFP as indicated. At 30 hpi, cells were processed in one of three major ways.

#### Fixatives

Cells were fixed with either 4%, 3%, 1% PFA, 3%, or 1% formaldehyde in PBS pH 7.4 (the latter prepared from a formalin stock containing trace methanol) for 20 min at RT, or pre-chilled 100% methanol or ethanol for 20 min, mounted to slides in 5 μl SlowFade Gold (Life Technologies), and sealed with nail polish.

#### Permeabilization methods

As indicated, cells were first fixed with 4% PFA for 20 min at RT, then incubated with just PBS (untreated) or pre-chilled 0.2% (Fig 4B and 4C), 0.1% Tx-100, 0.2% Saponin on ice for 10 min, ice-cold 1:1 mix of methanol and ethanol for 5 min on ice, or 2 mg/mL, 1 mg/mL, or 0.5 mg/mL of Zwittergent 3–12 (all detergents in PBS) for 1 min on ice, mounted to slides in 5 μl SlowFade Gold (Life Technologies), and sealed with nail polish.

Samples were double-blinded and viewed on an Axioskop 2 (Zeiss) inverted widefield fluorescence microscope or an Axio Observer Z1 (Zeiss) (Fig 4B and 4C) with a 63X 1.4 NA oil objective (Zeiss) and the frequency of inclusions containing ER-RFP intraluminal structures was quantified in 50–100 cells for each experiment. Values from three independent experiments were averaged and standard errors were calculated. Statistically significant differences were assessed by a one-way ANOVA followed by Dunnett's Multiple Comparison *post hoc* analysis comparing each condition to untreated with a p-value < 0.05 considered significant. Statistics and graphs were prepared with Prism (GraphPad Software) and Photoshop CS6 (Adobe).

### Imaging and Quantitation of ER-RFP Structures within Fixed *Coxiella burnettii* Pathogenic Vacuoles

HeLa cells grown on glass coverslips to 50% confluence were infected with *Coxiella burnetii* Nine Mile RSA439 (phase II, clone 4) and synchronized by centrifugation (3,000 rpm for 30 min at 10°C). At 24 hpi, cells were transfected with ER-RFP. At 52 hpi, cells were fixed with 4% paraformaldehyde (PFA) in PBS for 20 minutes at RT, incubated with 1 μg/mL Hoescht 33258 (Life Technologies) in PBS for 20 minutes at RT, mounted to slides in 5 μl SlowFade Gold (Life Technologies), and sealed with nail polish. Images were captured on a Leica SP5 laser scanning confocal inverted microscope equipped with a 100x 1.4 NA oil objective and over 50 infected cells were assessed for the frequency of ER-RFP structures within the pathogenic vacuole. Images were minimally processed with Photoshop CS6 (Adobe) for presentation.

## Supporting Information

S1 Fig Fixation-induced intralumenal ER-RFP structures within inclusions persist through many post-fixation manipulations. HeLa cells were infected with *C*. *trachomatis* LGV L2 and transfected with ER-RFP for 30 hr, fixed with 4% paraformaldehyde, treated with the indicated permeabilization solutions, and assessed for the frequency of ER-RFP within inclusions. Treatments included the nonionic detergent Triton X-100 (Tx-100), Saponin, an amphipathic glucoside, a 1:1 mix of methanol and ethanol, or Zwittergent 3–12, a dipolar ionic detergent for various times. 50–100 inclusions were enumerated in each experiment, and the mean ± SEM for three independent experiments is shown. * indicates P < 0.05 by one-way ANOVA and Dunnett's Multiple Comparison *post hoc* analysis comparing each condition (gray bars) to the control (black bars).
(TIF)

S1 Movie Z-sections through a fixed *C*. *trachomatis* infected cell expressing ER-RFP and Sec61β-GFP at 30 hpi. (AVI)

S2 Movie Z-sections through a living *C*. *trachomatis* infected cell expressing ER-RFP and Sec61β-GFP at 30 hpi. (AVI)

S3 Movie QuickTime Virtual Reality file of 3D volume of ER-RFP within a fixed cell infected with *C*. *trachomatis* for 30 hr. (MOV)

S4 Movie QuickTime Virtual Reality file of 3D volume of Sec61β-GFP within a fixed cell infected with *C*. *trachomatis* or 30 hr. (MOV)

S5 Movie QuickTime Virtual Reality file of 3D volume of ER-RFP within a living cell infected with *C*. *trachomatis* for 30 hr. (MOV)

S6 Movie QuickTime Virtual Reality file of 3D volume of Sec61β-GFP within a living cell infected with *C*. *trachomatis* for 30 hr. (MOV)

S7 Movie Time-lapse video microscopy of living *C*. *trachomatis* infected cells expressing ER-RFP undergoing chemical fixation. White arrowheads in first frame indicate *C*. *trachomatis* inclusions. Time is displayed in each frame as min:sec.
(AVI)
