# Supplementary figures and images for: Differential Translocation of Host Cellular Materials into the Chlamydia trachomatis Inclusion Lumen during Chemical Fixation
Source: PLoS One. 2015 Oct 1;10(10):e0139153. doi: 10.1371/journal.pone.0139153 (PMC4591358; doi:10.1371/journal.pone.0139153)

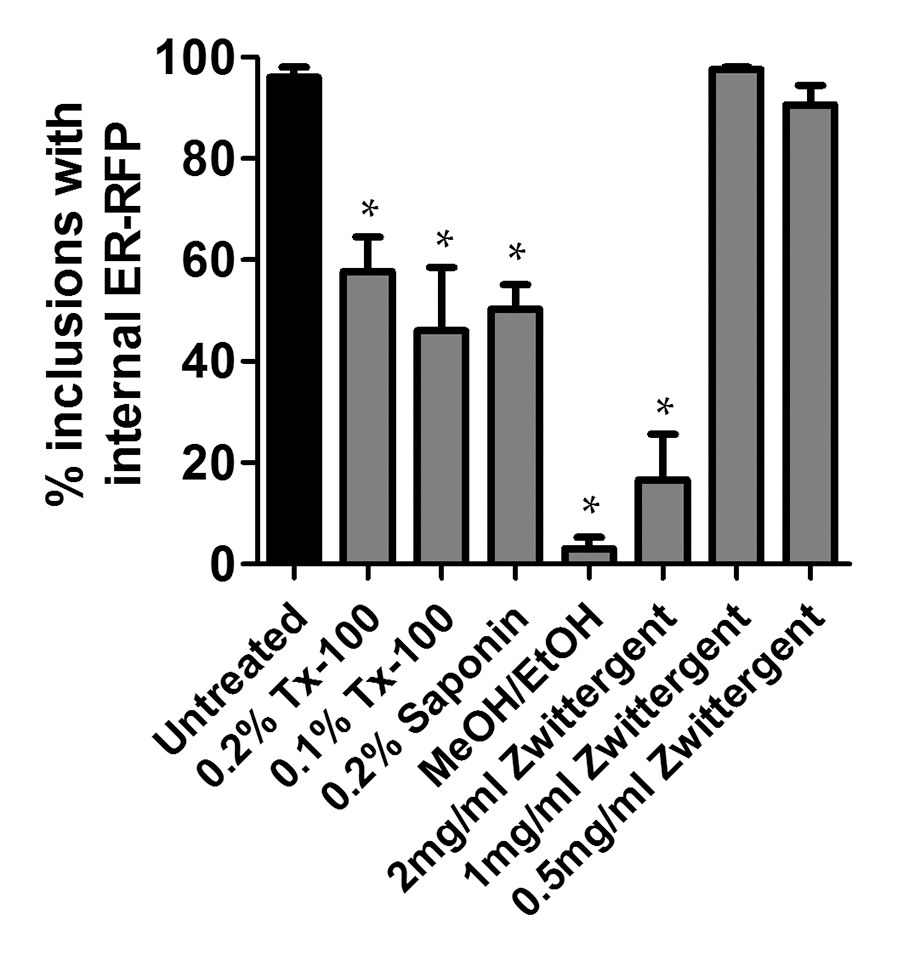

Supplement: S1 Fig — HeLa cells were infected with C. trachomatis LGV L2 and transfected with ER-RFP for 30 hr, fixed with 4% paraformaldehyde, treated with the indicated permeabilization solutions, and assessed for the frequency of ER-RFP within inclusions. Treatments included the nonionic detergent Triton X-100 (Tx-100), Saponin, an amphipathic glucoside, a 1:1 mix of methanol and ethanol, or Zwittergent 3–12, a dipolar ionic detergent for various times. 50–100 inclusions were enumerated in each experiment, and the mean ± SEM for three independent experiments is shown. * indicates P < 0.05 by one-way ANOVA and Dunnett's Multiple Comparison post hoc analysis comparing each condition (gray bars) to the control (black bars). (TIF) [file pone.0139153.s001.tif]
